# Diverse roles of epidermal growth factors receptors in oral and cutaneous canine melanomas

**DOI:** 10.1186/s12917-020-2249-2

**Published:** 2020-01-29

**Authors:** Emerson Soares Veloso, Ivy Nayra Nascimento Gonçalves, Tatiany Luiza Silveira, Fernando Soares Oliveira, Déborah Soares Vieira, Geovanni Dantas Cassali, Helen Lima Del Puerto, Enio Ferreira

**Affiliations:** Department of General Pathology, Institute of Biological Sciences, Federal University of Minas Gerais, Belo Horizonte, MG Brazil

**Keywords:** Melanoma, EGFR’s, Dog, Immunohistochemistry

## Abstract

**Background:**

The epidermal growth factor receptors participate in the physiological processes such as regulation of morphogenesis, proliferation and cell migration, but when overexpressed or overactivated they may play an important role in neoplastic progression. Melanoma is the most aggressive skin neoplasm and is characterized by elevated invasion and low survival rates in both humans and dogs. In human melanomas the overexpression of EGFR, HER3 or HER4 is associated with poor prognosis. In canine melanomas the epidermal growth factor receptors expression has not been evaluated. Therefore, this study evaluated the expression of epidermal growth factor receptors by immunohistochemistry and investigated their relationship with morphological characteristics and proliferative indices in cutaneous and oral canine melanoma.

**Results:**

In cutaneous melanoma an increased proliferative index was associated with increased cytoplasmic HER4 and reduced EGFR and HER3 protein expression. In oral melanomas, membranous HER2 protein expression correlated with occurrence of emboli, but *ERBB2* gene amplification wasn’t observed.

**Conclusion:**

Thus, our work evidenced the relationship between HER4 and the stimulus to cell proliferation in cutaneous melanomas, in addition to the relationship between HER2 and the occurrence of emboli in oral melanomas.

## Background

The epidermal growth factor receptors EGFR, HER2, HER3 and HER4 participate in the regulation of cell morphogenesis, proliferation, migration and adhesion [1]. These receptors are transmembrane proteins composed of an extracellular epidermal growth factor (EGF)-binding domain, a transmembrane domain and a cytoplasmic domain with tyrosine kinase activity [2]. EGF binding leads to receptor dimerization, tyrosine kinase activation and the direct or indirect activation of signaling pathways such as Ras/MAPK, PLCγ1/PKC, Akt and STAT, which subsequently stimulate cell proliferation and differentiation [3, 4].

In various types of neoplasms, overexpression of these receptors is mainly associated with neoplastic progression and poor prognosis [5]. The main factor underlying this overexpression is gene amplification. Amplification of the *ERBB* genes and polysomy of chromosome 7—where the EGFR gene is located—are correlated with poorer prognosis in human melanoma [6].

EGFR, HER3 and HER4 overexpression is correlated with poorer prognosis in human melanoma [7, 8]. In particular, EGFR overexpression is associated with decreased survival rates and increased tumor size [6, 9]. In addition, EGFR expression is higher in metastases compared with primary tumors [10]. In vitro and in silico studies in human melanoma cell lines and experimental murine melanoma models found that epidermal growth factor receptors are promising therapeutic targets [11, 12].

Canine melanoma is characterized by high invasive and metastatic potential and is considered one of the most aggressive forms of skin cancer in dogs [13]. The canine disease shares clinical, histological and molecular characteristics with human melanoma [14]. Prognosis is poorer when lesions are located on the mouth or toes; the 1-year survival rate in these instances is only 10%, even when surgery and chemotherapy are performed [13, 15]. The factors involved in genesis of canine melanoma are still controversial [16]. No study has investigated the expression of epidermal growth factor receptors in canine melanoma.

Studies performed with experimental murine melanoma models found that cetuximab, a monoclonal antibody that inhibits EGFR activity, reduces tumor invasion and suppresses metastasis formation [11, 17]. However, there are no descriptions of this treatment in dogs.

Due to the high aggressiveness of canine melanoma and the scarcity of information of association with epidermal growth factor receptors and canine melanoma progression, the aims of the present study were to characterize the expression of EGFR, HER2, HER3 and HER4 in skin and oral canine melanoma and to determine their relationship with the histopathological characteristics and proliferative indices of these tumors.

## Results

### Histological characteristics

We analyzed 76 cases diagnosed as melanoma. During the pigmentation evaluation 16 received score 0 (amelanotic), 29 score 1, 13 score 2 and 18 score 3. After excluding the pigmentation cases 3, we obtained a total of 58 cases that were included in this study, of which 34% (20/58) were oral lesions and 66% (38/58) skin lesions.

In the oral samples, 90% (18/20) presented histologically as epithelioid and 10% (2/20) as fusiform. Ulcers were observed in 80% (16/20) and desmoplasia in 30% (6/20). Junctional activity was present in 59% (10/17; in 3 cases it was not possible to assess junctional activity due to extensive involvement of the epidermis by ulcers) and emboli in 50% (10/20).

In the cutaneous samples, 68% (26/38) presented histologically as epithelioid and 32% (12/38) as fusiform. Ulcers were observed in 39% (15/38) and desmoplasia in 47% (18/38). Junctional activity was present in 27% (10/37; in 1 case it was not possible to assess junctional activity due to extensive involvement of the epidermis by ulcers) and emboli in 50% (19/38).

### Expression of epidermal growth factor receptors

**Fig. 1:**
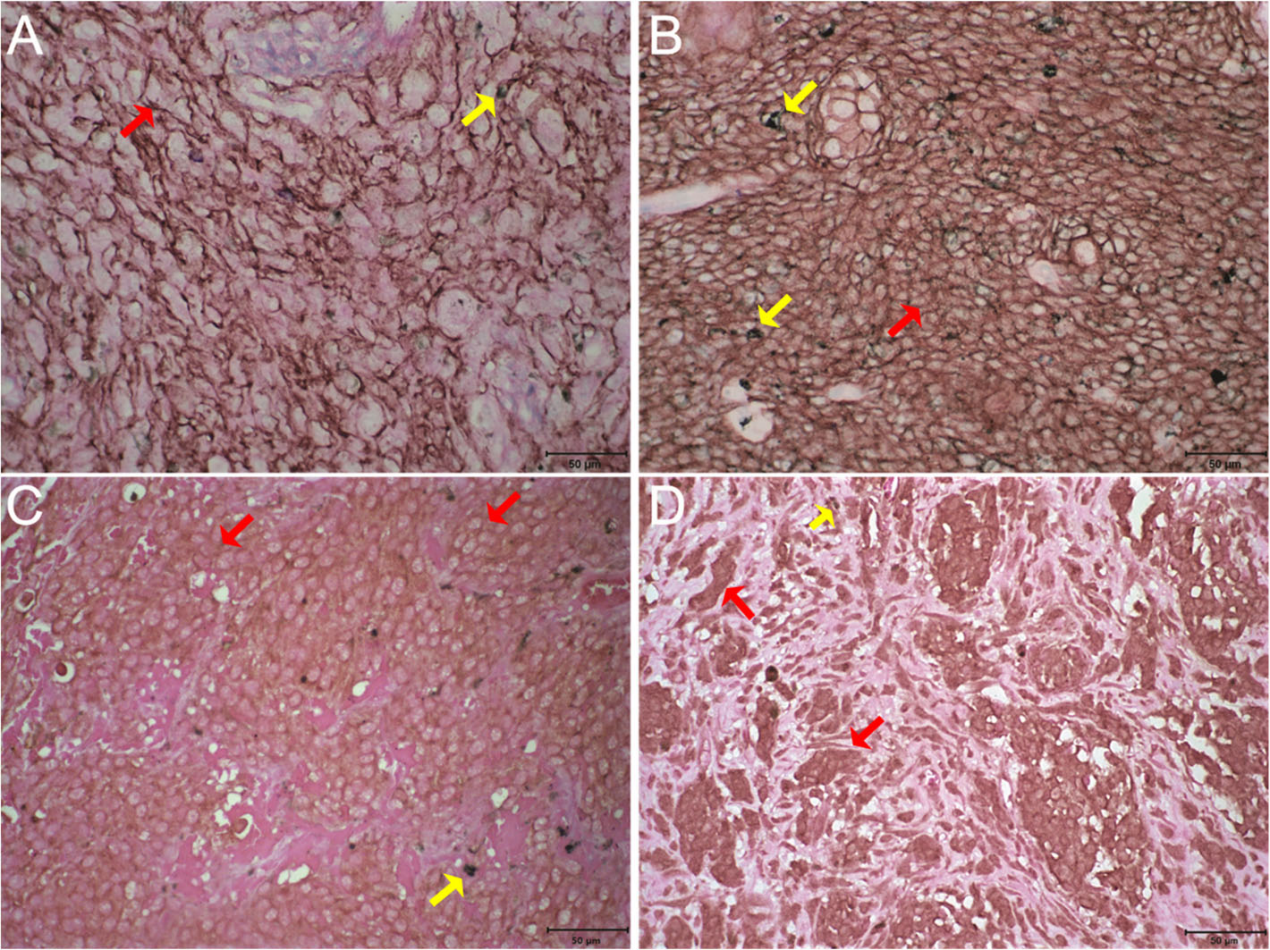
EGFR and HER2 immunohistochemistry in canine melanoma. **a**) Membrane immunostaining 3+ for EGFR in cutaneous melanoma. **b**) Membrane immunostaining 3+ for EGFR in oral melanoma. **c**) Membrane immunostaining 2+ HER2 in cutaneous melanoma. **d**) Cytoplasmatic immunostaining moderate in more 30–60% of the neoplastic cells for HER2 in oral melanoma. Bar: 50 μm. Red arrows: immunostaining. Yellow arrows: Melanin

**Fig. 2:**
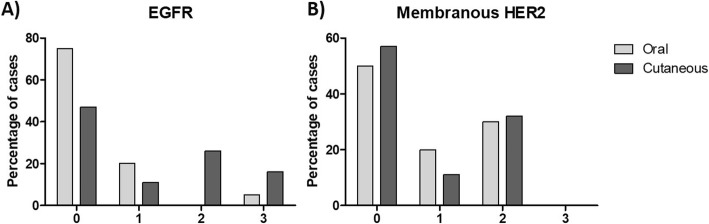
Immunohistochemical expression of EGFR and HER2. **a**) Percentage distribution by EGFR expression score in oral and cutaneous melanomas. **b**) Percentage distribution by HER2 expression score in oral and cutaneous melanomas

EGFR expression (Fig. 1a and b) was detected in 25% of the oral and 53% of the skin tumors. Receptor overexpression (score 3) was identified in only 5 and 16% of lesions, respectively (Fig. 2a).

Membranous HER2 expression (Fig. 1c) was detected in 50% of the oral and 43% of the skin tumors; no cases received a score of 3 (Fig. 2 b). Cytoplasmic HER2 (Fig. 1d) expression was not detected in 30% of oral lesions and in 35% of skin lesions.

**Fig. 3:**
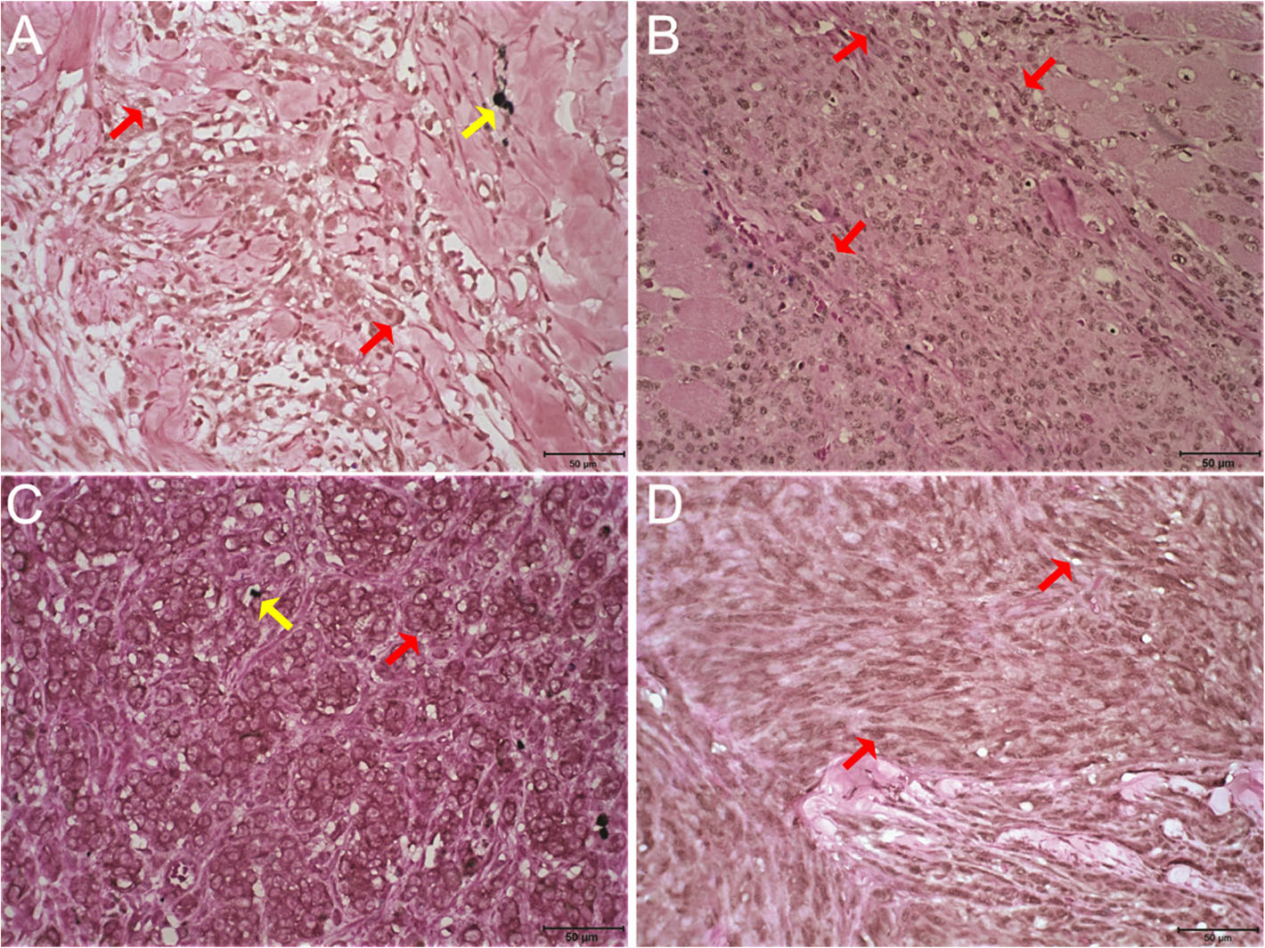
HER3 and HER4 immunohistochemistry in canine melanoma. **a**) Cytoplasmatic moderate (2+) immunostaining for HER3 in cutaneous melanoma. **b**) Nuclear moderate (2+) immunostaining for HER3 in oral melanoma. **c**) Membrane immunostaining moderate (2+) for HER4 in cutaneous melanoma. **d**) Nuclear and cytoplasmatic immunostaining weak (1+) for HER4 in oral melanoma. Bar: 50 μm. Red arrows: immunostaining. Yellow arrows: Melanin

**Fig. 4:**
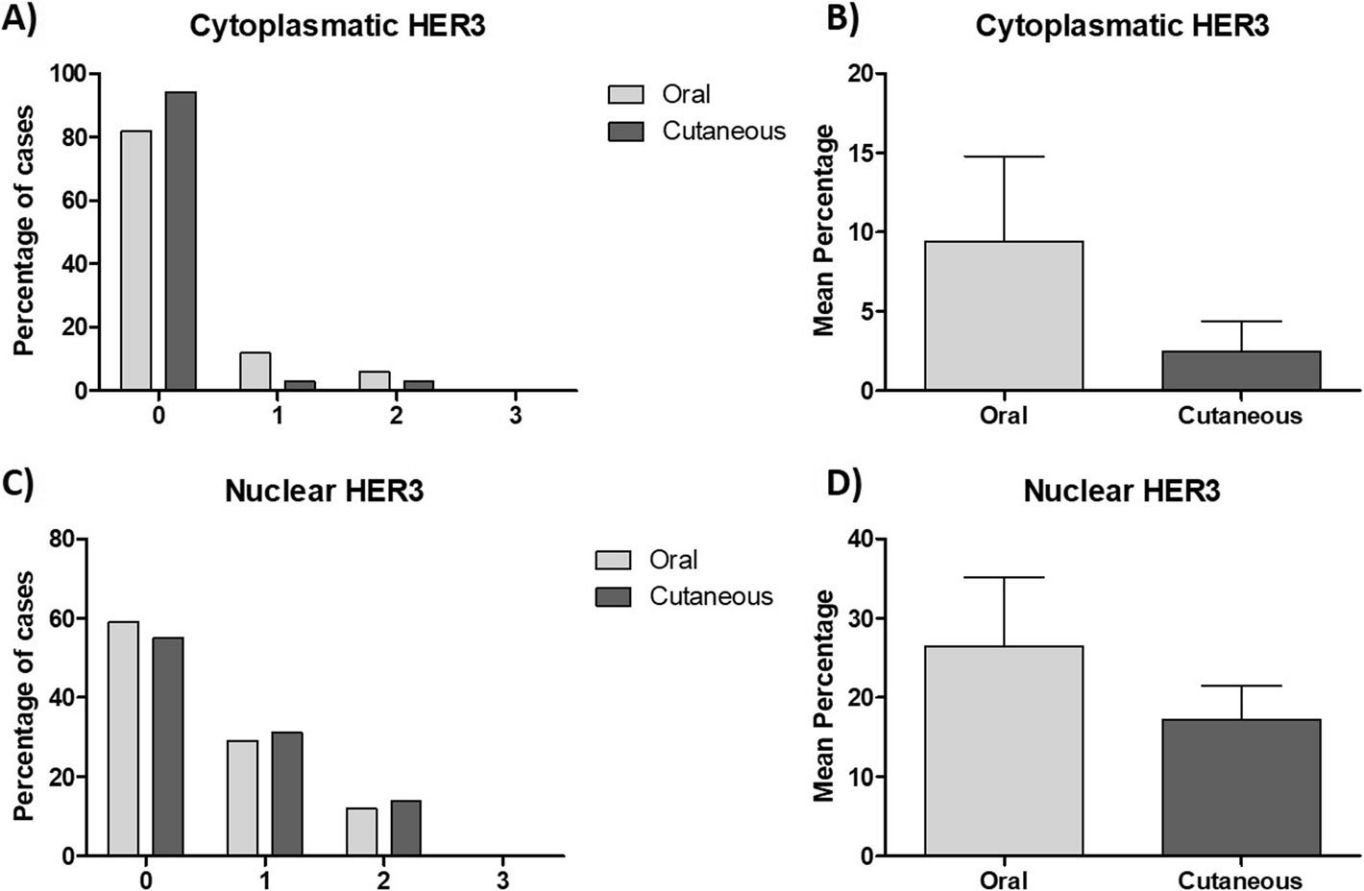
Immunohistochemical expression of HER3. **a**) Percentage distribution by HER3 cytoplasmatic expression score in oral and cutaneous melanomas. **b**) Mean percentage of cytoplasmic HER3 expression in neoplastic cells of oral and cutaneous canine melanomas. **c**) Percentage distribution by HER3 nuclear expression score in oral and cutaneous melanomas. **d**) Mean percentage of nuclear HER3 expression in neoplastic cells of oral and cutaneous canine melanomas

Membranous HER3 was not detected in any sample. Cytoplasmic expression (Fig. 3a) was observed in only 18% of the oral and 6% of the skin tumors (Fig. 4a); the mean percentage of labeled cells was 9 and 3% in the oral and skin tumors, respectively (Fig. 4b). Nuclear HER3 expression (Fig. 3b) was detected in 41% of the oral and 44% of the skin lesions (Fig. 4c); the average percentage of labeled cells was 26 and 17% in the oral and skin tumors, respectively (Fig. 4d).

**Fig. 5:**
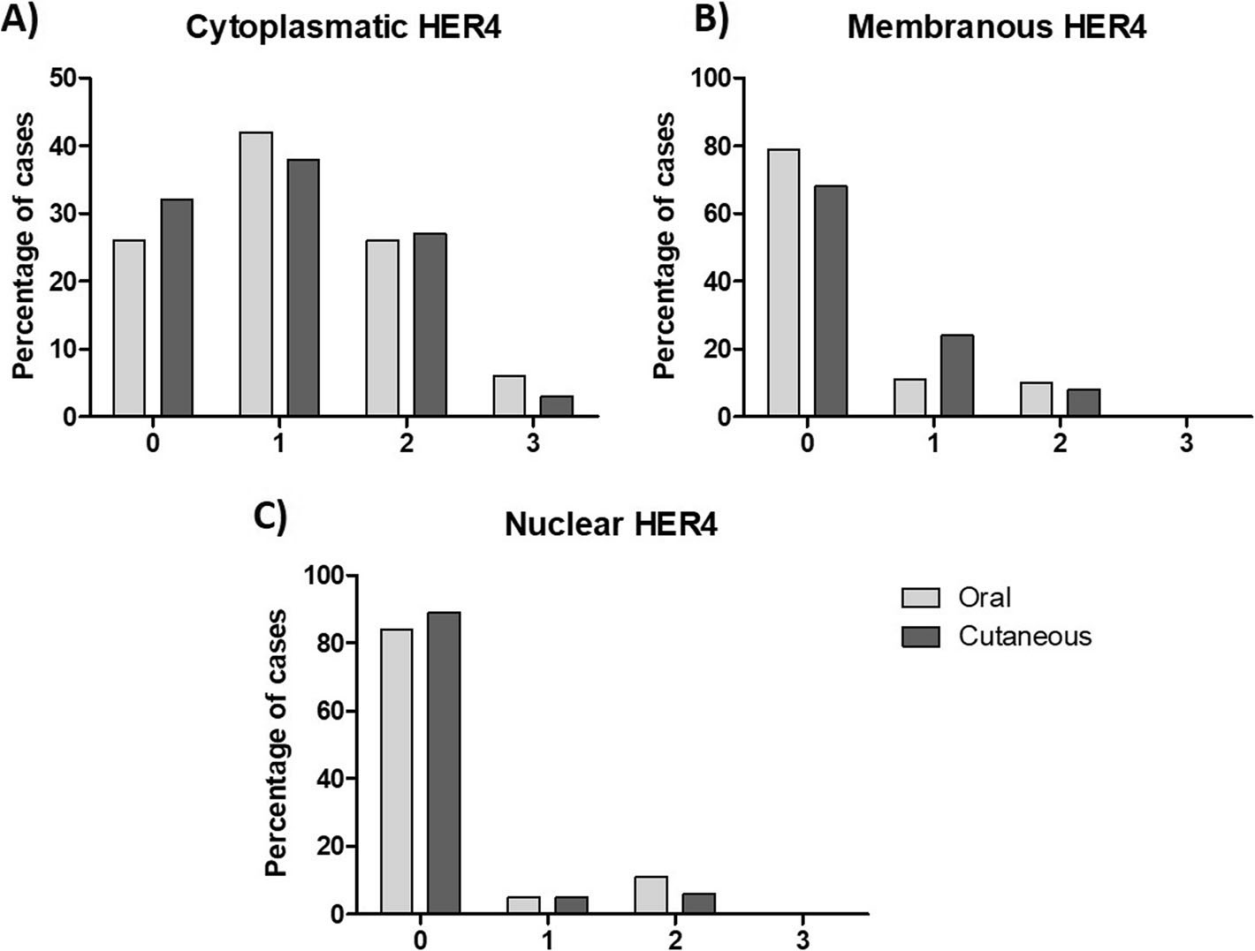
Immunohistochemical expression of HER4. Percentage distribution by HER4 score expression (**a**) cytoplasmatic; (**b**) membranar and (**c**) nuclear in oral and cutaneous canine melanomas

Cytoplasmic HER4 expression (Fig. 3d) was weak (1+) in 42% of the oral and 38% of the skin tumors (Fig. 5a). Membranous expression (Fig. 3c) was observed in 21% of the oral and 68% of the skin lesions (Fig. 5b). Nuclear expression (Fig. 3d) was detected in only 16% of the oral and 11% of the skin neoplasms (Fig. 5c).

### Cell proliferation and epidermal growth factor receptor expression

The mean mitotic index was 4.71 ± 2.00 and 3.56 ± 2.87 for oral and skin melanomas, respectively. There was no statistically significant difference between the oral and skin melanomas, nor was there any association between the mitotic index and growth factor receptor labeling.

The Ki-67 expression was used to calculate a mean proliferative index of 22.06 ± 15.09 and 32.30 ± 18.18 for skin and oral melanomas, respectively. There was no statistically significant difference between the samples from each site.

In the skin samples, an increased proliferative index correlated with increased cytoplasmic HER4 (r: 0.4510; p: 0.0308) and reduced EGFR (r: − 0.4468; p: 0.0326) and nuclear HER3 (r: − 0.5465; p: 0.0070) expression. The proliferative index was lower in lesions with desmoplasia (r: − 0.4232; p: 0.0442). In the oral lesions, an increased proliferative index correlated with the epithelioid variant (r: 0.5698; p: 0.0421).

### Relationship between epidermal growth factor receptors and histological characteristics

Higher EGFR expression correlated with decreased cytoplasmic HER2 expression (r: − 0.3357; p: 0.0422) in the skin melanomas. This association was not detected in oral tumors.

Membranous HER2 expression correlated with diagnoses of the epithelioid versus the fusiform variant (r: 0.3851; p: 0.0186) as well as increased nuclear HER3 expression (r: 0.3806; p: 0.0241) in skin lesions. In oral melanomas, membranous HER2 expression correlated with the occurrence of emboli (r: 0.4578; p: 0.0487) as well as cytoplasmic HER3 expression (r: 0.6720; p: 0.0031). In both tumor sites, there was a correlation between membranous HER2 and membranous HER4 expression (r: 0.4669; p: 0.0041, for the skin and r: 0.4885; p: 0.0338, for the oral tumors). Cytoplasmic HER2 expression was associated with lower cytoplasmic HER4 expression in oral melanomas (r: − 0.5291; p: 0.0198).

Cytoplasmic HER3 expression was associated with increased membranous HER4 expression in both skin (r: 0.3981; p: 0.0179) and oral (r: 0.5012; p: 0.0404) lesions. Additionally, cytoplasmic HER4 expression was associated with the epithelioid variant (r: 0.4235; p: 0.0090) and desmoplasia (r: 0.4958; p: 0.0018) in skin tumors and with the epithelioid variant in oral lesions (r: − 0.4643, p: 0.0452).

### ERBB2 gene amplification

**Fig. 6:**
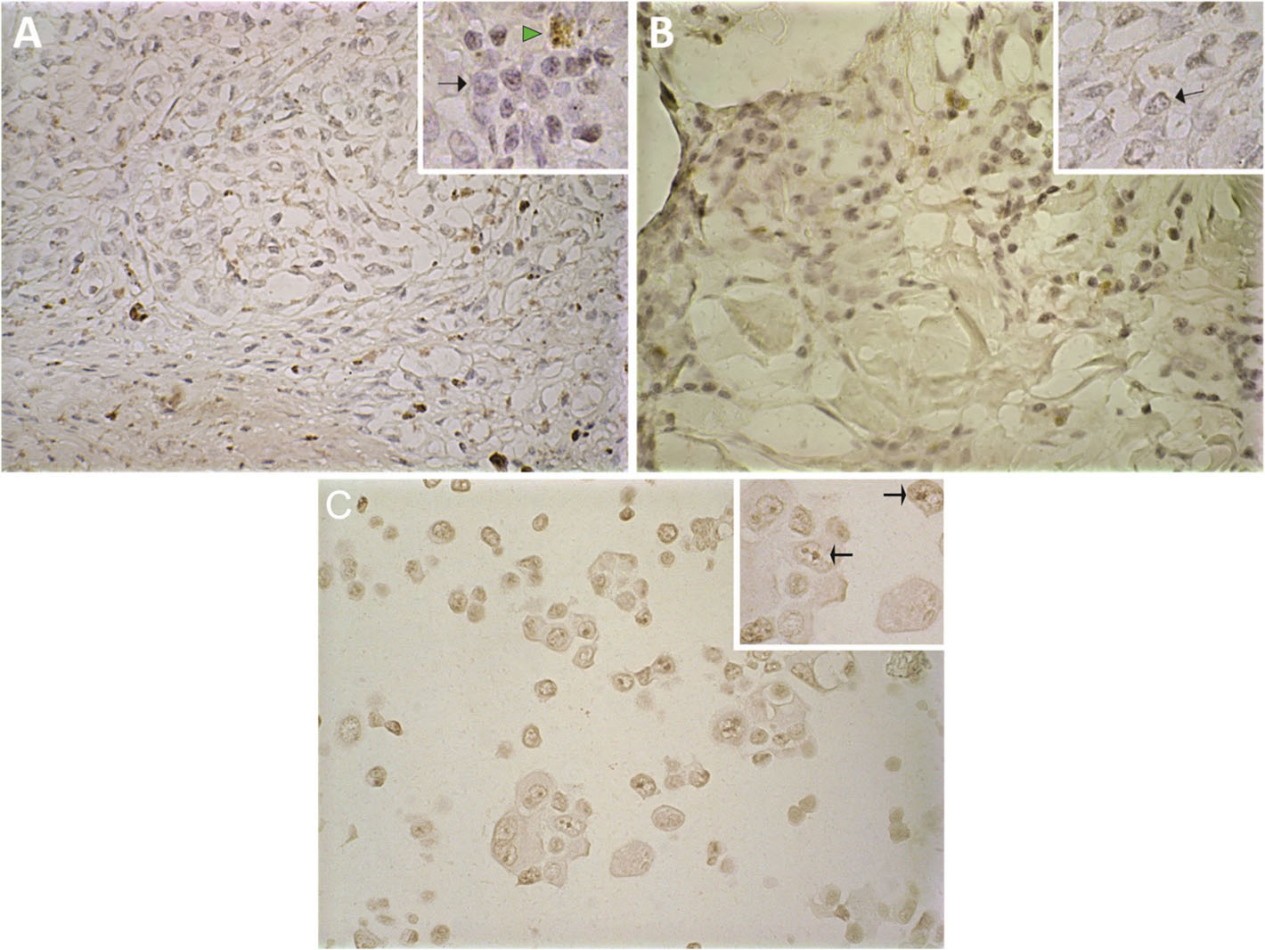
Chromogenic in situ hybridization (CISH) for *ERBB2* in canine melanoma. **a**) Cutaneous canine melanoma with normal expression (2–4 copies per cell) for *ERBB2*. **b**) Oral canine melanoma with normal expression (2–4 copies per cell) for *ERBB2*. **c**) Human mammary cancer cell line (positive control) with large clusters of *ERBB2* in cancer cell nuclei. Arrows: Presence of chromogen. Arrow Head: melanin

CISH (Chromogenic in situ hybridization) was performed on four oral (one with HER2 immunohistochemical expression) and nine skin (five with HER2 immunohistochemical expression) melanomas. All of the tested samples exhibited up to four gene copies (nuclear spots) and thus were classified as having normal gene expression (Fig. 6 a and b).

## Discussion

As with human melanoma, canine melanoma often exhibits poor response to conventional chemotherapeutic agents [15] as well as reduced survival and high metastasis rates, especially for oral lesions [13]. In this paper we report for the first time, as far as we know, the expression of EGFR, HER2, HER3, and HER4 in canine melanomas.

Due the highest number of pigmented cells, samples that receive score 3 for pigmentation were not included in this study. Because it is a melanocytic neoplasm, it is well known that amelanotic melanomas represent less differentiated neoplasms. However, amelanotic melanomas do not necessarily represent a worse prognosis, since the association between pigmented tumors and survival is controversial. Some authors described an association between longer survival and pigmented tumors for both oral and skin [18, 19], but others described there is no significant association between the absence of pigments and shorter survival [20].

In our samples, the proliferative index was correlated with growth factor receptors expression. In melanomas, Ki67 is a better prognostic marker than the mitotic index and had a significant impact of prognosis by multivariate analyses [21]. This correlation was not observed with mitotic index, probably because during mitosis the signaling of growth factor receptors is altered due to the changes of the endocytosis process, which is slower than usual [22]. Besides that, the stimuli of growth factors are only necessary in the earlier stages of cell cycle progression [23].

Our data demonstrate a relationship between increased cytoplasmic HER4 expression and a high proliferative index in skin melanoma. HER4 is a receptor consisting of a ligand-binding ectodomain, a transmembrane, juxtamembrane and cytoplasmatic tyrosine kinase regions [24]. At least 2 isoforms are described for the juxtamembrane domain (JMa and JMb) [25] and cytoplasmatic domain (CYT-1 and CYT-2) [26]. The HER4 proteolytic processing, induced by binding of growth factors such as neuregulin (NRG) and heparin-binding epidermal growth factor (HB-EGF), involves a cleavage event, mediated for metalloproteinase tumor necrosis factor-α-converting enzyme (TACE or ADAM17), in JMa isoform [27], and a subsequent cleavage, mediated for presenilin, resulting in the releases into the cytosol, and posterior translocation for the nuclei or accumulation within mitochondria and endoplasmic reticulum, of the cytosolic HER4 fragment [28–30]. The cytoplasmic domain of HER4 can interact with PI3K/AKT pathway, which leads to cell proliferation [31]. The presence of cytoplasmic HER4 that we observe can be an indicative of cleavage and accumulation of cytosolic HER4 fragment, which could promote cell proliferation stimulation pathways, justifying the association found between HER4 cytoplasmic expression and proliferative index.

The cytoplasmic HER4 isoform was associated with poorer prognosis when detected in the lymph nodes of human patients with metastatic melanoma [32]. With regards to primary tumors, Zhu et al. (2018) recently demonstrated cytoplasmic HER4 expression in sinonasal mucosal melanoma and showed that it is an independent marker of poorer prognosis [33]. Mutations in the *ERBB4* gene—which encodes HER4—were associated with oncogenic gain-of-function effects in human melanoma [8]. We found a direct relationship between membranous HER4 expression and both membranous HER2 and cytoplasmic HER3 expression. Associations between epidermal growth factor receptors, which facilitate heterodimerization, are important for tyrosine kinase activity amplification and/or reconstitution and for ligand affinity [34]. As a result, cytoplasmic HER4 expression in canine melanoma is associated with parameters of poorer prognosis, such as a high proliferative index.

HER3 expression was mainly observed in the nuclei of neoplastic cells and was negatively correlated with the proliferative index in skin melanomas. Membranous HER3 expression—which was not detected in the present study—was found to be a marker of poorer prognosis in in vitro and in vivo studies of human melanoma [7]. Conversely, nuclear HER3 expression is considered an indicator of good prognosis, since it has been associated with longer survival in human melanoma [35]. These findings corroborate our results, which demonstrate a correlation between nuclei expression and reduction of the proliferative index. However, although nuclear HER3 expression was associated with better prognosis, some evidence indicates that the translocation of this receptor into the nucleus is the result of a mechanism related to EGFR inhibitor resistance [36]. Some authors have found that HER3 nuclear translocation exclusively occurs in cells that co-express other epidermal growth factor receptors [37]. Therefore, while nuclear HER3 expression seems to be an indicator of good prognosis in both canine and human melanoma, its translocation is probably not an indicator of cellular resistance but of co-expression of other epidermal growth factor receptors.

We further identified a correlation between nuclear and cytoplasmic HER3 and membranous HER2 expression. HER3 is the only epidermal growth factor receptor family member considered to be an inactive pseudo-kinase, as it lacks a functional tyrosine kinase activity domain. For this reason, heterodimer formation is necessary for this receptor to become functional [38]. This phenomenon explains the relationship found between HER2 and HER3 in the analyzed model. It also indicates that the HER2-HER3 interaction might facilitate HER3 activation as well as cell differentiation in canine melanoma, as this receptor does not appear to be related to the stimulation of proliferation in the present study.

Membranous HER2 overexpression and *ERBB2* gene amplification did not occur in any of the samples analyzed in the present study. Nevertheless, the score of membranous expression was associated with the occurrence of emboli in oral tumors. Three different models of melanoma dissemination, where in all of them the participation of emboli occurs, are found in the literature [39]. 1) sequential model, melanoma spreads first to the sentinel lymph node, via lymphatic system, and then to the distant sites, via the bloodstream [40]; 2) simultaneous model, dissemination via lymphatic system and the bloodstream occurs at the same time [41]; and 3) differential metastasis model, the ability to metastasize is variable among melanomas. Some tumors have the ability to disseminate by both pathways (lymphatic and bloodstream), while some metastasize for only one of them [42, 43]. In canine melanomas, Milanta et al., has shown that the presence of emboli is associated with reduced survival [44].

HER2 activation leads to increased expression and stability of kinase Src, which plays an important role in regulating of migration and invasion [45]. Src can promote the expression of metalloproteinases (MMP’s) 2, 7 and 9, contributing to the degradation of extracellular matrix [46–48], besides compromising cell-cell adhesion by phosphorylating the E-cadherin/B-catenin complex leading to loss cell adhesion which stimulates the expression of transcription factors inducing epithelium-mesenchyme transition, thus favoring the invasion and formation of metastases [49, 50]. The results of studies investigating HER2 expression in human melanoma are controversial. Similar to our findings, Shayanfar et al., 2015 [51] and Kluger et al., 2004 [52] did not detect membranous overexpression or gene amplification. These findings suggest that HER2 is not useful as a prognostic biomarker in melanoma. Similar results were also described in a study published by Potti et al., who observed membranous HER2 overexpression in only 0.9% of the analyzed samples [53]. Conversely, Bodey et al. did identify HER2 overexpression in human melanoma [54]. One limiting factor for HER2 studies in human melanoma is the small number of cases. While our data are partially similar to reports in the literature indicating that HER2 has no prognostic value, they also indicate a relationship with embolus occurrence and thus with neoplastic progression.

Membranous EGFR overexpression was detected in a few cases, and the presence of this receptor was associated with a reduced proliferative index in skin lesions. This finding contradicts observations made in human melanoma, where EGFR participates in the stimulation of melanocyte proliferation [55] and is associated with poorer prognosis [17]. Thus, in canine melanoma, EGFR might be associated with other functions, such as cell differentiation and autophagy [56, 57].

## Conclusion

This is the first time HER2, HER3, Her4, and EGFR are reported in canine melanoma. In summary, we identified a relationship between the proliferative index and both increased cytoplasmic HER4 expression and reduced EGFR and HER3 expression in skin melanomas. Moreover, we established a relationship between membranous HER2 expression and the occurrence of emboli, even in the absence of *ERBB2* gene amplification. Therefore, we conclude that among the epidermal growth factor receptors HER4 and HER2 are probably those associated with canine melanoma progression, HER4 being associated with proliferation stimulating pathways and HER2 associated with invasion stimulating pathways.

## Methods

### Ethical issues

The present study complied with the ethical principles for animal experimentation and was approved by the animal ethics committee (*Comissão de Ética no Uso de Animais*–CETEA/CEUA) of the Federal University of Minas Gerais (*Universidade Federal de Minas Gerais*–UFMG) (ruling no. 008/2016).

### Specimens selection and Histopathological evaluation

Were analyzed cases of melanoma in dogs of different breeds, obtained from the collection of Laboratory of Comparative Pathology, Institute of Biological Sciences, UFMG. The samples were sent to the Laboratory of Comparative Pathology for histopathological diagnosis after surgical resection at the Veterinary Hospital of UFMG and at the Department of Veterinary Medicine, Federal University of Lavras. The samples used in this study were collected between 2005 and 2017 and fixed in 10% neutral buffered formalin and following a 24-h fixation the specimens were subjected to macroscopic evaluation, sectioned and processed using the same routine paraffin embedding technique, stained with hematoxylin-eosin for analysis under a light microscope and histological classification (epithelioid or fusiform) according to the World Health Organization system [58].

All analyses were performed under a conventional light microscope Olympus–BX41 at 40x magnification - high-power field - (HPF). The tumors were qualitatively classified based on their pigmentation according to a 0-to-3 scoring system as follows: absence of pigmented neoplastic cells (0); 1–25% of pigmented neoplastic cells (1); 26–50% of pigmented neoplastic cells (2); and ≥ 51% of pigmented neoplastic cells (3). Tumors that received a score of 3 were not included in the present study due to difficulties in visualizing cytoplasmic immunohistochemical staining. Tumors with a score of 0 were categorized as amelanotic and tumors with a score of 1 or 2 were categorized as melanotic. Other prognostic factors assessed included the presence of emboli, ulceration, desmoplasia and junctional activity (lentiginous or pagetoid spread), classified as 0 (absence) or 1 (presence), and mitotic index, calculated as the mean number of cells undergoing mitosis in 10 HPF. A high mitotic index was defined as ≥4 for oral lesions and ≥ 3 for skin lesions, based on [59].

Immunohistochemistry for Anti-Melan-A and anti-Melanoma antigen (PNL-2) antibodies were used to confirm the diagnosis of melanoma in all analyzed samples, and the entire slide was analyzed. Immunohistochemical analysis for Melan-A was performed in all samples, and for Melanoma Antigen was performed for all amelanotic tumors, as well as for melanotic tumors that did not exhibit Melan-A labeling. Positive cases were defined as those with cytoplasmic labeling in more than 10% of the neoplastic cells [59].

### Immunohistochemistry

Immunohistochemical staining was performed, by manual technique and all samples for each antibody were performed at the same time, according to the peroxidase reaction method with a polymerized secondary antibody (Advance™ HRP; Dako North America; Via Real Carpinteria, CA, USA or Novolink Polymer Detection System; Leica Biosystems, Newcastle upon Tyne, UK or Histofine® Simple Stain MAX PO (MULTI), Nicherei Fresh Inc., Chun, Tokyo, Japan) for identification. Antigen retrieval was performed by means of humid heat (water bath at 98 °C) or pressurized humid heat at 125 °C (Pascal® Pressure Cooker; Dako Cytomation, Glostrup, Denmark) with Target Retrieval Solution Citrate - pH 6.0 (Dako Cytomation, Glostrup, Denmark). Only for EGFR immunodetection, the antigen retrieval method used was the enzymatic recovery in pepsin (400 mg dissolved in 99 ml H_2_O and 1 ml HCl 1 N) at 37 °C for 30 min. To block endogenous peroxidases, slides were incubated twice for 10 min in a 3% H_2_O_2_ solution in methyl alcohol. To block endogenous proteins, slides were incubated for 20 min with Protein Block Serum-Free Ready to Use (Dako North America; Via Real Carpinteria, CA, USA). Slides were incubated with primary antibodies for 30 min (anti-HER3) or 16 h (anti-EGFR, anti-HER2, anti-HER4, anti-Ki-67, anti-Melan-A and anti-Melanoma Antigen (PNL-2). Following a 1-min incubation with the chromogen 3′3-diaminobenzidine (Liquid DAB + Substrate Chromogen system; Dako North America, Via Real Carpinteria, CA, USA), the sections were counterstained with Giemsa stain (1:5) for 30 min and then rinsed with a hydrochloric acid solution (1:100), absolute alcohol and finally isopropyl alcohol for 1 min. Through this treatment and the subsequent counterstaining, the melanic pigment acquires a greenish hue that is different from the brownish hue of chromogen reaction with DAB/primary antibody. Table 1 lists the manufacturers, clones, dilutions and incubation times for all antibodies used.

**Table 1:** IHC protocol for EGFR, Her-2, Her-3, Her-4, Ki67, Melan-A, Melanoma antigen antibodies

Antibody	Manufacturer	Clone	Dilution	Incubation time (h)	Antigen retrieval	Amplification
EGFR	Invitrogen	31G7	1:50	16 h	Pepsin^a^	Advance HRP
Her-2	Dako	Polyclonal	1:200	16 h	Citrate buffer + WB^b^	Advance HRP
Her-3	Genetex	Polyclonal	1:100	30 min	EDTA + WB^b^	Histofine
Her-4	Santa Cruz	C18	1:100	16 h	Citrate buffer + Pascal^c^	Histofine
Ki-67	Dako	MIB-1	1:50	16 h	Citrate buffer + Pascal^c^	Novolink
Melan-A	Dako	A103	1:100	16 h	Citrate buffer + Pascal^c^	Novolink
Melanoma Antigen	Santa Cruz	PNL-2	1:100	16 h	Citrate buffer + Pascal^c^	Novolink

^a^Stove (37 °C)

^b^WB – Water bath (98 °C)

^c^Pascal – Water bath (125 °C)

For the negative control, the primary antibody incubation step was omitted and replaced by incubation with immunoglobulins from the same species as the primary antibody was made. We also tested the replacement of the primary antibody with the antibody diluent used (Antibody Diluent with Background Reducing Components; Dako North America; Via Real Carpinteria, CA, USA).

### Interpretation of Immunohistochemical findings

All histological assessments were performed under a conventional light microscope at 40x magnification (Olympus–BX41). For Anti-Melan-A and PNL-2 antigen positive cases were defined as those with cytoplasmic labeling in more than 10% of the neoplastic cells [59].

The anti-Ki-67 antibody was used to calculate the proliferative index of lesions based on the percentage of nuclear-labeled cells out of 500 counted cells at the area of hot spot (relatively dense concentration of positive cancer nuclei). A high proliferative index was defined as ≥19.5% for oral tumors [20] and ≥ 15% for skin tumors [19].

EGFR, HER2, HER3 and HER4 immunohistochemical analysis was performed at the entire slide. EGFR and HER2 expression on the neoplastic cell membrane was categorized according to the following scoring system (Additional file 1: Fig. S1): (0) very weak incomplete membrane labeling in less than 10% of cells; (1+) very weak incomplete labeling in more than 10% of cells; (2+) weak or moderate incomplete membrane labeling in more than 10% of cells or strong complete labeling in less than 10% of cells; (3+) strong complete membrane labeling in more than 10% of cells (adapted from the Wolff et al., 2013 [60]). We further analyzed cytoplasmic HER2 expression; the results were categorized as a function of the percentage of immunolabeled cells: (0) no labeling; (1+) cytoplasmic labeling in less than 10% of neoplastic cells; (2+) cytoplasmic labeling in 10 to 30% of neoplastic cells; (3+) cytoplasmic labeling in 30–60% of neoplastic cells; and (4+) cytoplasmic labeling in more than 60% of neoplastic cells.

Nuclear, cytoplasmic and membranous HER3 expression was separately investigated in neoplastic cells and categorized according to the following scoring system: (0) no labeling; (1+) weak; (2+) moderate; and (3+) strong, based on the labeling intensity of at least 30% of cells [61]. In addition, we estimated the percentage of labeled cells in a semiquantitative manner.

Nuclear, cytoplasmic and membranous HER4 expression was separately investigated in neoplastic cells and categorized according to the following scoring system: (0) no labeling; (1+) weak; (2+) moderate; and (3+) strong, based on the labeling intensity of at least 10% of cells [62].

### Chromogenic in situ hybridization (CISH)

CISH was performed to investigate the number of chromosome 9 and *ERBB2* gene copies using the CISH SPOT–Light Chromogenic ISH detection kit (Invitrogen Corporation, Camarillo, CA, USA). 4 μm histological sections of the selected lesions, that is positive for HER2 expression in immunohistochemistry, were hydrated and pretreated according to the manufacturer’s instructions. Next, the slides were incubated with a HER2 DNA probe (SPOT-Light HER2 Probe) in a Dako Hybridizer for 5 min at 90 °C and 10 h (overnight) at 37 °C. The hybridized probe was detected using the CISH Polymer Detection Kit II, and the tissues were counterstained with Mayer’s hematoxylin for 10 s. Previously tested human breast samples were used as positive controls. The performance of the CISH was performed according to the manufacturer’s criteria and previous standardizations of our group [63].

Analysis and interpretation of the hybridization results were performed based on the recommendations supplied by the kit manufacturer. For hybridization analyses, 30 neoplastic cells were counted under a light microscope at 40x magnification. Absence of amplification was defined as 1 to 4 gene copies per nucleus in more than 50% of neoplastic cells; low amplification was defined as 5–10 copies or the presence of small clusters in each nucleus in more than 50% of neoplastic cells; and high amplification was defined as more than 10 copies or large clusters in each nucleus in more than 50% of neoplastic cells. Clusters corresponding to the presence of the gene are restricted to the nucleus, with well-defined staining, whereas pigmentation is observed in the cytoplasm, with granular and intense staining. CISH was performed in 13 samples, four oral melanomas (one with HER2 immunohistochemical expression) and nine cutaneous melanomas (five with HER2 immunohistochemical expression).

### Statistical analysis

Statistical analyses were performed with GraphPad Prism v. 5.0 (GraphPad Software, La Jolla, CA, USA). Relationships between variables were investigated with the Chi-square or Fisher’s exact test. Possible correlations were evaluated using a Spearman’s or Pearson’s test. For quantitative results, means were compared using a t-test or the Mann-Whitney test depending on the normality of data distribution. The significance level for associations and correlations was set to *p* ≤ 0.05.

## Supplementary information

**Additional file 1: Figure S1.** EGFR or HER2 immunohistochemistry in canine melanoma. A) Absence of membrane immunostaining for HER2 in cutaneous melanoma. B) Membrane immunostaining 1+ for HER2 in cutaneous melanoma. C) Membrane immunostaining 2+ for HER2 in cutaneous melanoma. D) Membrane immunostaining 3+ for EGFR in oral melanoma.

## Data Availability

The datasets used and/or analysed during the current study are available from the corresponding author on reasonable request.

## Abbreviations

CISH: (Chromogenic in situ hybridization)
EGF: (Epidermal growth factor)
HPF: (High-power field)
