# Supplementary figures and images for: Diverse roles of epidermal growth factors receptors in oral and cutaneous canine melanomas
Source: BMC Vet Res. 2020 Jan 29;16:24. doi: 10.1186/s12917-020-2249-2 (PMC6988198; doi:10.1186/s12917-020-2249-2)

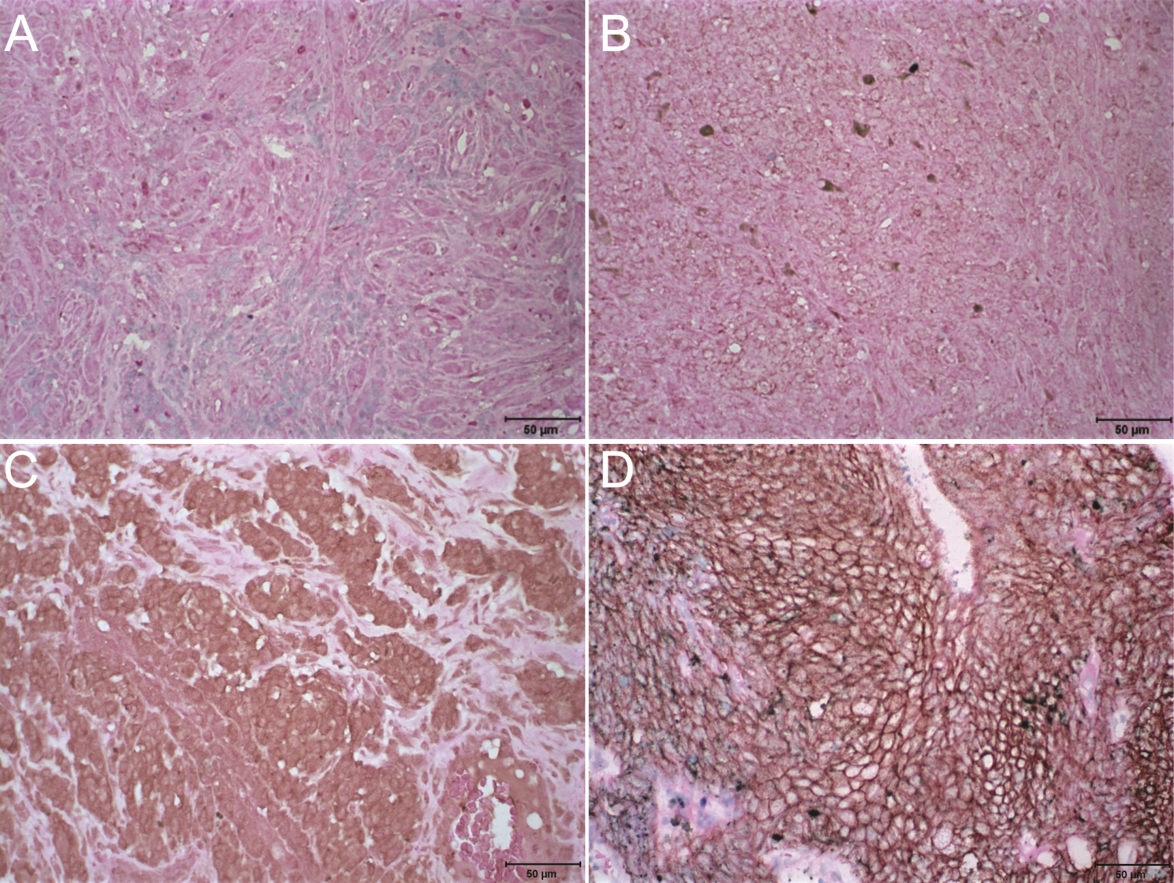

Supplement: Supplementary file 1 — Additional file 1: Figure S1. EGFR or HER2 immunohistochemistry in canine melanoma. A) Absence of membrane immunostaining for HER2 in cutaneous melanoma. B) Membrane immunostaining 1+ for HER2 in cutaneous melanoma. C) Membrane immunostaining 2+ for HER2 in cutaneous melanoma. D) Membrane immunostaining 3+ for EGFR in oral melanoma. [file 12917_2020_2249_MOESM1_ESM.tif]
